# An iEEG Recording and Adjustable Shunt-Current Conduction Platform for Epilepsy Treatment

**DOI:** 10.3390/bios12040247

**Published:** 2022-04-15

**Authors:** Changhua You, Lei Yao, Pan Yao, Li Li, Ping Ding, Shuli Liang, Chunxiu Liu, Ning Xue

**Affiliations:** 1 State Key Laboratory of Transducer Technology, Aerospace Information Research Institute (AIR), Chinese Academy of Sciences, Beijing 100190, China; youchanghua18@mails.ucas.ac.cn (C.Y.); yaopan19@mails.ucas.edu.cn (P.Y.); cxliu@mail.ie.ac.cn (C.L.); 2 School of Electronic, Electrical and Communication Engineering, University of Chinese Academy of Sciences, Beijing 100049, China; 3 School of Microelectronics, Shanghai University, Shanghai 200444, China; yaol@shu.edu.cn; 4 SPF Biotechnology Co., Ltd., Beijing 102100, China; leeli1960@163.com; 5 Functional Neurosurgery Department, Beijing Children’s Hospital, Capital Medical University, Beijing 100045, China; dingpingwy@126.com (P.D.); liangsl_304@sina.com (S.L.); 6 Personalized Management of Chronic Respiratory Disease, Chinese Academy of Medical Sciences, Beijing 100190, China

**Keywords:** neural electrophysiological signal recording, current conduction treatment, conduction electrode, temporal lobe epilepsy

## Abstract

This paper proposes a compact bioelectronics sensing platform, including a multi-channel electrode, intracranial electroencephalogram (iEEG) recorder, adjustable galvanometer, and shunt-current conduction circuit pathway. The developed implantable electrode made of polyurethane-insulated stainless-steel materials is capable of recording iEEG signals and shunt-current conduction. The electrochemical impedance of the conduction, ground/reference, and working electrode were characterized in phosphate buffer saline solution, revealing in vitro results of 517.2 Ω@1 kHz (length of 0.1 mm, diameter of 0.8 mm), 1.374 kΩ@1 kHz (length of 0.3 mm, diameter of 0.1 mm), and 3.188 kΩ@1 kHz (length of 0.1 mm, diameter of 0.1 mm), respectively. On-bench measurement of the system revealed that the input noise of the system is less than 2 μVrms, the signal frequency bandwidth range is 1 Hz~10 kHz, and the shunt-current detection range is 0.1~3000 μA with an accuracy of above 99.985%. The electrode was implanted in the CA1 region of the right hippocampus of rats for the in vivo experiments. Kainic acid (KA)-induced seizures were detected through iEEG monitoring, and the induced shunt-current was successfully measured and conducted out of the brain through the designed circuit-body path, which verifies the potential of current conduction for the treatment of epilepsy.

## 1. Introduction

Epilepsy is a chronic neurological disorder of the brain that affects people of all ages. Approximate 50 million people currently suffer this disorder [1]. The incidence of epilepsy is about 0.3 to 0.5%, while the prevalence is about 5 to 10 people per 1000 [2]. Current therapies for epilepsy are mainly based on anti-epilepsy drugs, surgical treatment, and neuromodulation to prevent the occurrence of seizures [3,4,5]. During antiepileptic drug therapy, more than 30% of patients with epilepsy do not respond adequately to antiepileptic drug therapy and 20% of patients still suffer from repeated seizures [6]. Although some patients benefit from resection surgery, a substantial proportion of patients are not suitable candidates for conventional surgical management and some patients refuse a craniotomy due to the prevalence of complication [7]. Moreover, resection surgery can achieve a long-term favorable outcome for seizure control in only about 50% patients [8].

Electroceuticals are emerging therapies that acquire electrical neural signals and modulate the nervous system with stimulation treatment [9,10,11]. This strategy has promoted the development of implantable electrical probes, miniaturized neuro-recording and neurostimulation circuits, and has significant potential to treat a variety of stubborn and chronic diseases, such as Parkinson’s disease, heart failure, depression, spinal cord injury, etc. [12,13,14]. The clinical application of pacemakers, neural stimulation, artificial retinae, and vagus nerve stimulation has driven smart implantable electronic devices towards precision therapy [15]. Intracortical microstimulation (ICMS) based on an implanted neuroprosthesis can trigger gamma oscillations in the primary auditory cortex in a similar way to the natural communication process [16]. Neurostimulation has been employed in epilepsy treatment over the past decade. Typical neurostimulation therapies include deep brain stimulation (DBS), vagus nerve stimulation (VNS), and trigeminal nerve stimulation (TNS). Among them, electrical stimulators are used to suppress the onset of epilepsy by applying current to specific parts of the human brain, which has a proven safety profile and a satisfactory curative effect in epilepsy treatment [17]. Cukiert et al. treated nine patients with refractory temporal lobe epilepsy (TLE) via DBS at the hippocampus and achieved a 76~80% reduction in seizure frequency [18]. Over 100,000 VNS devices have been implanted worldwide (as of 2015) to treat epilepsy, with a typical stimulation range of 20~30 Hz for a duration of 30~90 s and an off-time of 5 min. Results showed a 50% reduction in seizures after 2~3 years of treatment [19]. TNS is an emergent therapy. Olivié et al. studied 40 patients that have been involved in TNS therapy and showed that 50% of the group presented a seizure frequency reduction at 12 months with a stimulation intensity of 6.2 mA [20]. However, the side effects of neurostimulation may include dysphonia, hoarseness, depression, obesity, and cough. As a blind device, the open-loop system cannot monitor the brain state or adjust the stimulation profile accordingly to optimize seizure control. Thus, more and more researchers are focusing on the design of closed-loop systems [21,22]. In recent years, some studies have demonstrated that the closed-loop strategy is an effective alternative to an open-loop neurostimulator because it improves the efficiency of neurostimulation and reduces the occurrence of possible adverse effects [21].

Temporal lobe epilepsy (TLE) is a common drug-refractory epilepsy. Monitoring the electroencephalogram (EEG) is an indispensable tool for the diagnosis and treatment of neurological diseases [6,23]. Based on long-term detection of intracranial electroencephalograms (iEEG), it was found that the focal epileptic discharge will generate a relatively high voltage (2000~3000 μV) in a localized region of the cortex at the initial onset of a seizure [6]. Furthermore, the seizure propagation time is much slower than normal neuro-electric activity and seizure propagation typical exhibits a relatively fixed diffusing pathway. Due to the side effects of traditional neurostimulation therapy, researchers further proposed a “conduction” mechanism as a passive method for seizure control and neuroprotection. In this method, the epileptic discharge is conducted from a relatively high voltage at the seizure focus to a low voltage region outside the brain using a conduction microelectrode. Contrary to traditional therapies with “inhibition” as the basic mechanism (i.e., anti-epilepsy drug, surgery, and neurostimulation), the electronic conduction treatment based on a “conduction” mechanism that has been preliminarily confirmed to reduce seizure frequency and hippocampal cell apoptosis in rats with Kainic acid (KA)-induced acute TLE [24].

As an emerging therapy for epilepsy, a compact system for current conduction treatment has been proposed. The system should have a closed-loop iEEG recorder and a shunt-current conduction pathway with an integrated amperemeter. The system would be used as a standard platform to monitor electrophysiological parameters and evaluate efficiency during shunt-conduction treatment. To conduct the intracranial local epileptic discharge to a lower voltage extracranial region, a novel electrode including ground, recording sites, and a current conduction port needs to be designed. Moreover, to examine the dose-response relationship between the conductive intensity and therapeutic outcomes of the current conduction treatment, an adjustable circuit system for the intensity of the shunt-current is also necessary. The system should provide real-time detection of iEEG with intervention in the initial seizure and detection of the density of the shunt-current flowing through the electric conduction pathway.

In this study, we aim to develop a novel platform for neural signal recording and shunt-current conduction for real-time epileptic seizure detection and epilepsy treatment based on the “conduction” mechanism. In addition, in contrast to conventional neurostimulation therapies, an animal experimental protocol for current conduction treatment has also been proposed. The proposed platform would quantitatively study and evaluate the curative effect of shunt-current conduction for epileptic seizure treatment.

This paper is organized as follows: Section 2 contains the design of the neural electrode, iEEG recording and shunt-current measurement system, and the setup of animal experiments. Section 3 provides measurement results in vitro and in vivo. Finally, Section 4 discusses the contributions of the paper and suggestions for future work.

## 2. Materials and Methods

Details on the compact bioelectronics sensing platform, including a multi-channel electrode, intracranial electroencephalogram (iEEG) recorder, shunt-current conduction circuit pathway, adjustable galvanometer, and the animal experimental design are provided as follows.

### 2.1. Design and Fabrication of the Neural Electrode

**Figure 1:**
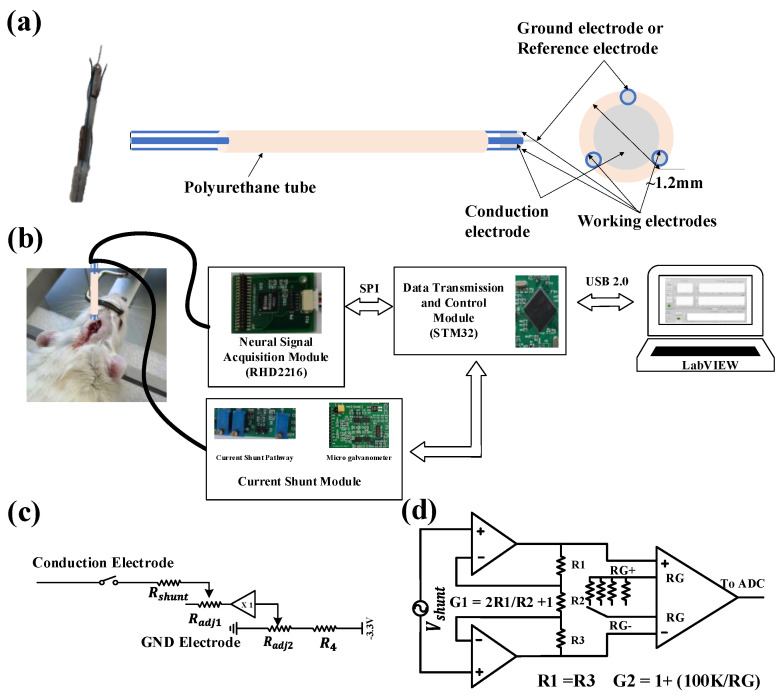
Developed system and protocol for recording the iEEG signal and shunt-current densityin a Sprague Dawley rat. (**a**) Schematics of the electrode design; (**b**) the architecture block diagram of a closed-loop adjustable shunt-current intensity circuit and system for current conduction treatment of epilepsy; (**c**) the circuit diagram for the current shunt module; (**d**) the circuit diagram for the micro galvanometer, where Vshunt is the voltage drop across the current sensing resistors (R_shunt1_ and R_shunt2_).

Neural electrodes with four contact sites were designed to conduct the shunt-current and record neural signals, as illustrated in Figure 1a. To reduce the electrochemical impedance, especially contact interface impedance between the electrode and the tissue, along the current conduction pathway, a customized spiculate conduction electrode with a stainless-steel needle head was designed and fabricated with a diameter of 0.8 mm and a length of 1 mm. Three filament electrodes (two working electrodes and one reference/ground electrode) were made of stainless-steel (diameter 0.1 mm) as contact material and polyurethane as the insulating material. The exposed contact site length of the filament electrode is 0.1 mm and 0.3 mm, respectively. The technical data for the neural electrode is summarized in Table 1. Lastly, the four electrodes were glued with a biocompatible epoxy and encapsulated in a polyurethane tube with a diameter of 1.2 mm approximately. The ground electrode and the reference electrode were combined into one contact site to minimize the number of electrodes and reduce the total volume of the neural electrode bunch. When the system is working in the conduction mode, a current loop is formed through the tissue, the conduction electrode, the circuit, and the ground electrode, such that the current flows from focal epileptic discharge sites to the outside of the brain. On the other hand, when the system is working in the iEEG signal acquisition mode, the ground electrode will work as a reference electrode together with the working electrodes to provide a voltage differential input for recording the iEEG signal.

### 2.2. iEEG Recording and Shunt-Current Measurement System

Figure 1b shows a schematic diagram of the experimental setup for the platform. The circuit system in the platform consists of a neural signal acquisition module, current shunt module, data transmission and control module, and a software interface module for real-time display, storage, and replay of historical signals. The fabricated probe is implanted in the rat brain, and the leads are connected to the recording module and current shunt module. The default mode of the system is iEEG signal recording. The dual-channel iEEG signal is transmitted to the host computer (PC) through a series port (USB2.0) for real-time display. When an epileptic seizure is detected by the simple threshold algorithm coded in MCU or artificial visual inspection, the system will turn off the iEEG signal recording mode and enable the shunt-current measurement mode. The current density can be transmitted to the PC through series communication. A head-stage was designed to expand the usability of the circuit system. Once combined with the neural electrodes and the epilepsy detection algorithm, a closed-loop current conduction treatment platform for epilepsy therapy was realized.

#### 2.2.1. iEEG Acquisition Module

Neural electrophysiological signals are typically classified into two categories: action potentials (AP) and local field potential (LFP). The depolarization of the membrane of a neuron generates extracellular AP, with waveforms in the range of 100 Hz to 10 kHz and a duration in the range of a few milliseconds. The other type of biopotentials known as LFP consists of lower-frequency neural waveforms in the range of 1 Hz to 200 Hz [25]. A special digital electrophysiology interface chip RHD2216 (Intan tech, Los Angeles, CA, USA) with a high internal common mode rejection ratio (CMRR) of the chip (up to 82 dB) was used in our front-end system for high performance iEEG signal acquisition. Since the frequency of the neural electrophysiological signal is mainly distributed below 1000 Hz, and interference of the DC polarization voltage exists, the frequency band of the system was set to 1 Hz~10 kHz and the sampling rate of the system was set to 25 kHz. After the neurophysiological signal is acquired by the neural probes, the signals are amplified, filtered, and digitized by RHD2216. In addition, electromagnetic shielding was adopted to suppress the influence of external electromagnetic interference (mainly the 50 Hz power line frequency).

#### 2.2.2. Current Conduction Module and Micro Galvanometer

Considering that a high shunt current may damage brain tissue, by adjusting the resistors in the current conduction path, the maximum shunt current was set to less than 3 mA after referring to design advice for implantable neurostimulation devices [26]. Figure 1c,d illustrates the functional block diagram for the current shunt module, which consists of control circuit and micro galvanometer. When the abnormal discharge activity of neuron is detected, the switch (Figure 1c) will be turned on and the shunt current path is connected. The intensity of the shunt-current can be adjusted by two high precision adjustable resistors (R_adj1_ and R_adj2_). Compared to electrical stimulation, current conduction treatment involves an electrical current output, not an afferent input. Therefore, a minimum potential (−150 mV) was assigned at the end of the current shunt path to prevent the shunt-current flowing into the brain. To detect current intensity, a micro galvanometer was designed. By measuring and amplifying the voltage drop across the current sensing resistors (R_shunt_) with a very low resistance (8.2 Ω), the current amplitude in the current conduction path can be calculated as I_shunt_ = V_shunt_/R_shunt_. The shunt-current is usually at the level of microamperes. Therefore, a two-stage amplifier structure consisting of two operational amplifiers and an instrumentation amplifier was used to measure Vshunt with a sub-mV amplitude. To acquire high current detection resolution over the wide shunt-current range, the total gain of micro galvanometer was adjusted by adjustable gain resistors (R_G_), using a four-to-one analog switch controlled by a DIP switch.

#### 2.2.3. Data Transmission and Control Module

The system used a STMicroelectronics microprocessor (STM32F407) as the core, which has an excellent working performance sufficient for the acquisition of dual-channel neurophysiological signals and shunt-current density. STM32F407 reads the converted digitalized neural signal from RHD2216 through an SPI interface and transmits the data to a PC through a USB 2.0 bus in synchronous transmission mode. To implement high-speed data collection and transmission, a double-buffer was adopted for real-time observation and analysis of the data. A timer interrupt mode was adopted to ensure data acquisition every 20 μs precisely, which is conducive to further research of the signal frequency spectrum analysis. Since both channels were used in the system, and only the data in one channel were converted per iteration, each individual channel is updated every 40 μs, giving an effective sampling rate of 25 kHz. On the other hand, when the current shunt module is turned on, the density of the shunt current can be obtained from the analog-digital-converter (ADC) and transmitted to the PC.

### 2.3. In Vivo Animal Experiments

A short-term animal experiment was conducted on adult male SD rats to verify the current conduction and recording capability of the designed compact platform, including both the fabricated probe and circuit. All animal procedures were approved by the Institutional Animal Care and Use Committee (IACUC) at SPF (Beijing) Biotechnology Co., Ltd., Beijing, China, and five adult male Sprague-Dawley (SD) rats (6–7 weeks, ~250 g) were used for the experiment. The rats were anesthetized with 1% pentobarbital sodium (40 mg/Kg), then fixed to a stereotaxic holder. After exposing the surface of the skull, an approximately 2 × 2 mm cranial window was drilled in the position of 2.2-mm *x*-axis, 3.5-mm *y*-axis, 3.2-mm *z*-axis from the bregma, where the fabricated probe was inserted into the brain. The two recording electrodes and conduction electrode were fixed in place. By simultaneously monitoring the recorded potential from the two electrodes, they were able to accurately reflect the signal potential at the exact location of the conduction site, and thus, accurately conduct the targeted current intervention. The dual-channel iEEG, sampling at a rate of 25 kHz, and the single channel shunt-current density, sampling at a rate of 200 Hz, were collected by the designed compact circuit system. Real-time data were displayed and stored with an application based on LabVIEW (National Instruments, Austin, TX, USA) and then processed in MATLAB (MathWorks, Natick, MA, USA). 2 μL Kainic acid (KA, 1 μg/mL) was unilaterally injected into the CA1 region of the right hippocampus within 5 min using a micro syringe to establish the rat epilepsy model. The injection needle was held in place for 3 min after completing the injection to allow diffusion of the KA. The rats were kept under anesthesia throughout the experiment and euthanized by CO_2_ suffocation after the experiment.

## 3. Results

### 3.1. In Vitro Characterization of the Neural Electrodes

**Figure 2:**
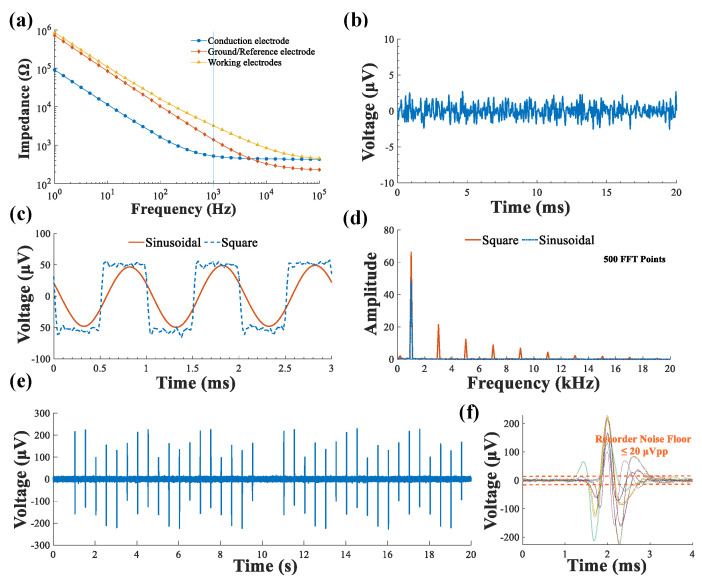
Developed system performance characterization. (**a**) In vitro impedance measurement of the electrodes with different sizes for the functional sites; (**b**) measured input-referred noise of the system; (**c**) measured output of the ±50 μV, 1 kHz square wave and the ±50 μV, 1 kHz sinusoidal wave; (**d**) measured output spectrum of the ±50 μV, 1 kHz square wave and the ±50 μV, 1 kHz sinusoidal wave; (**e**) measured output data segments of the designed iEEG signal acquisition system, where the recorder is fed with an emulated spike signal that is generated by a digital neural signal simulator; (**f**) the neural spikes signal and the recorder noise floor.

The performance of electrodes, especially for neural signal recording, is mainly determined by the electrochemical impedance between the electrode-tissue interface. Since the neural signal is as low as a few micro-volts, it is sensitive to the thermal noise, which is in proportion to the impedance [27]. The impedance of the shunt current conduction electrode needs to kept considerably low as well, to ensure the current is flowing outside the brain through the current conduction loop. The electrochemical impedance at the electrode-tissue electrolyte interface is inversely proportional to the effective electrode area. For this reason, considerable efforts have been adopted to increase the effective electrode areas for noise reduction. In this study, the electrical conduction electrode, working electrode, and ground electrode had an area of ~0.3 mm^2^, ~0.04 mm^2^ and ~0.1 mm^2^, respectively. Figure 2a shows representative electrochemical impedance spectra (EIS) for the electrodes measured in phosphate buffered saline (PBS). As expected, there was a negative correlation between electrode size and impedance since larger electrodes exhibited less impedance; the current conductance electrode had the largest exposed area (to conduct current from brain to the circuit) and the lowest impedance. The impedance decreased as the voltage frequency increased, which is also expected from the resistor/capacitor model for electrochemical impedance [27]. The conduction electrode had a measured impedance of 517.2 Ω@1 KHz, and the single channel of the filament electrode with an exposed length of 0.1 mm and 0.3 mm had a measured impedance of 1.374 kΩ@1 KHz, 3.188 kΩ@1 KHz, respectively.

### 3.2. On-Bench System Characterization

In signal detection, the input noise of an iEEG recording system must be far less than the amplitude of the signal to be measured. Figure 2b is the result for the equivalent input noise (EIN) as the system input is shorted. Its peak-peak voltage was VPP = 6~7 μV, and the root-mean-square voltage was Vrms = 1.26 μV. Thus, ideally, the system is able to detect iEEG signals greater than the Vpp (SNR > 1). To verify the system’s ability to detect weak signals, a square and a sinusoidal wave with an amplitude of 50 μVpp at 1 kHz(generated by an ultra low distortion function generator, Model DS360, Sunnyvale, CA, USA) were fed through the input of the signal acquisition module of the system. The output result shown in Figure 2c reveals that the system was able to restore the weak input signal in the passband range. Figure 2d illustrates the frequency spectrum of the measured square wave and sinusoidal from Figure 2c. It can be seen that the sinusoidal wave was successfully restored with negligible distortion, and the restored square wave signal had rich harmonics with a significant low noise floor. Extracellular spikes were then emulated by a digital neural signal simulator (Blackrock Microsystems, Salt Lake City, UT, USA). Figure 2e shows the measured data for the emulated neural spike signals over 20 s, where obvious neural spikes were detected. This indicates the signal acquisition module of the compact system is capable of measuring and restoring primitive neural spikes. The noise floor of the system after automatic filtering was lower than 20 μVpp (Figure 2f), which meets the requirements for iEEG signal acquisition. Overall, all results indicate our system has an excellent detection capability for weak signals in the passband (1~10,000 Hz) with low signal distortion and a low noise floor.

**Figure 3:**
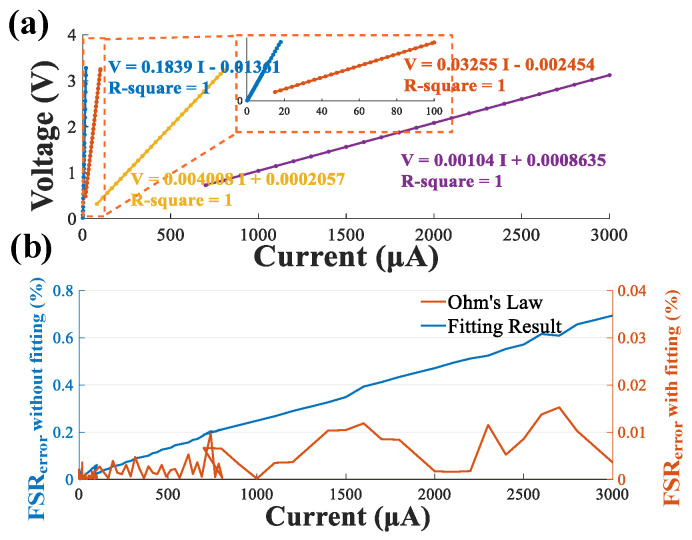
Validation of the micro galvanometer. (**a**) The linear correlation between the standard current and the output voltage measured by micro galvanometer; (**b**) the measured full-scale error (%*FSR_error_*).

To exam the function of the designed micro galvanometer module of the compact system, the chronopotentiometry mode of an electrochemical workstation (CHI 760e, CH Instruments, Shanghai, China) was adopted to generate a standard current in the range of 0.1~3000 μA, covering the whole range of current amplitudes for current conduction treatment. As described above, the galvanometer module has a wide adjustable current detection range by adjusting the gain resistor (RG) controlled by a four-to-one analog switch, so the system has high current detection sensitivity with a low full scale range error in each sub-range. To calibrate the micro galvanometer module, first the output voltage of the galvanometer module was recorded with a standard current as input in the ranges of 0.1~18 μA, 17~98 μA, 90~780 μA, and 750~3000 μA. The calibration result curve for the measured voltage as output and standard current as input generated by the electrochemical workstation is shown in Figure 3a. From the experiment results, the detection range of the designed micro galvanometer was 0.1~3000 μA with a remarkable linear response.

In the second step, the shunt-current value was calculated from the measured voltage drop across the current-sensing resistors based on Ohm’s law. The relationship between the calculated shunt-current and the standard current was further analyzed through an advanced fitting method to minimize the inevitable calibration differences caused by resistance accuracy and ADC error. The fitting method requires that a large amount of experimental data is collected in advance, then the predicted current formula is obtained by a one-dimensional linear method as follows:

(1) $$\begin{array}{l} I_{calculated(0.1-18)}=5.438\;\times \;U+0.07423\;(\mu A)\\ I_{calculated(17-98)}=30.72\;\times \;U+0.07542\;(\mu A)\\ I_{calculated(90-780)}=249.5\;\times \;U\;-\;0.05123\;(\mu A)\\ I_{calculated(750-3000)}=961.5\;\times \;U-\;0.8301\;(\mu A) \end{array}$$

Full-scale range error (%*FSR_error_*) is adopted to evaluate the accuracy of the developed micro galvanometer as follows:

(2) $$\%FSR_{error}=\frac{I_{calculated}-I_{ideal}}{I_{max}-I_{min}}\times 100\%$$

where *I_calculaed_* denotes the calculated current value, *I_ideal_* denotes the standard current value, and *I_max_ − I_min_* denotes the measurable range of the micro-current meter. As shown in Figure 3b, the maximum %*FSR_error_* for the four sub-ranges were 0.004%, 0.002%, 0.01%, 0.015%, respectively. The full-scale error was significantly low, revealing a high current measurement accuracy (>99.985%) for the system.

### 3.3. Acute In Vivo iEEG Recording and Current Conduction Experiment

**Figure 4:**
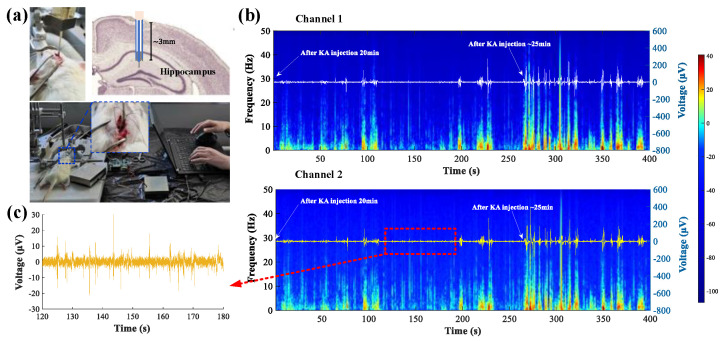
Acute in vivo neural signal recording experiment. (**a**) Photo image of animal experiment setup; (**b**) dual-channel iEEG signals and time frequency spectra after KA injection; (**c**) epileptic wave (Channel 2).

Following the animal protocol described in Section 2.3, a short-term animal experiment was conducted on adult male SD rats to verify the current conduction and recording capabilities of the designed compact platform, including both the fabricated probe and circuit. KA was injected into the CA1 region of the hippocampus of a rat to establish the acute temporal lobe epilepsy model, and the electrode tube was inserted in the area where KA was injected, as depicted in Figure 4a. Figure 4b represents the measurement results with dual-channel iEEG signals and the comprehensive time frequency spectrograms, starting at 20 min after injection of 2 μL KA. During the recording, an epileptic wave in the hippocampus was obviously induced (Figure 4c), and ictal discharge started at about 25 min after the KA injection, as shown in Figure 4b. This phenomenon was characterized by significantly increased numbers of high-amplitude LFPs. When the ictal events occurred, the signal amplitude was increased over the entire frequency range and multi-unit bursts frequently replaced single units. This is consistent with neural signal analysis of KA-induced TLE in the literature [28]. Data from the two channels in Figure 4b exhibited a similar signal profile over time. As ictal events occurred, the signal amplitudes were higher than 200 μV, which is at least four times higher than the normal neural signal. Thus, a threshold for the signal peak value had been set. Current conduction was turned on as the amplitude of the neural signal peak exceeded the threshold. The results indicate the system is capable of yielding stable neural signal readings. The total noise floor of the in vivo measurement systems was less than 10 μV.

**Figure 5:**
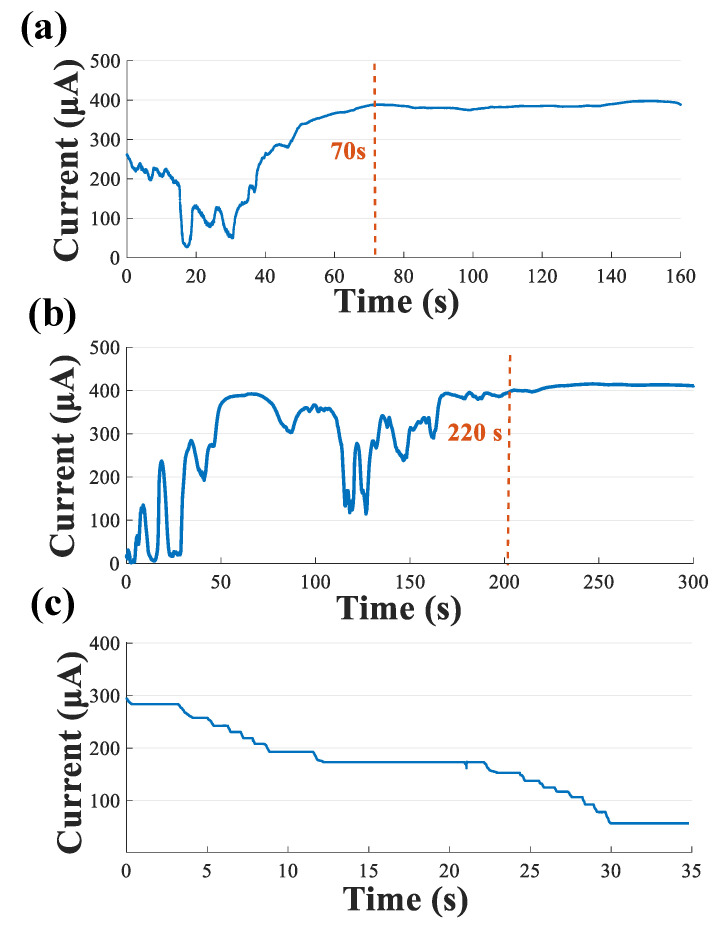
Acute in vivo current conduction experiment. (**a**) The shunt-current density measured in the range of 750–3000 μA; (**b**) the shunt-current density measured in the range of 90–780 μA; (**c**) the shunt-current density when the terminal potential was adjusted.

The fabricated electrode was implanted in the CA1 region of the right hippocampus. The current shunt module was turned on, thereafter. The shunt current thus flows from a relatively high voltage in the brain focus to the low voltage region outside the brain (−150 mV @ PCB) via a conduction microelectrode. Figure 5a shows the measured shunt-current density using a 3000 μA scale range. During the shunt-current recording period, the shunt-current density strongly fluctuated after the current conduction electrode had been implanted and the current started to conduct. However, the current stabilized at approximately 400 μA ~70 s after electrode implantation, which can be explained by continuous discharging at a stable stage. The shunt-current density measurement result from the 780 μA scale range had the same trend, as shown in Figure 5b. However, it reached a stabilization state at about 220 s with a magnitude of ~400 μA, which is consistent with the measurements made using a 3000 μA scale range. When the shunt-current density was stable, the current value could be adjusted by adjusting the terminal potential, as shown in Figure 5c. The terminal potential in the current conduction circuit was adjusted from −150 mV to −30 mV, gradually, which decreased the shunt-current value. At present, there are no studies on shunt-current conduction from the intracranial region to the outside of brain, so the mechanism remains to be explored.

## 4. Discussion

Current condition treatment as a neuromodulation therapy for epilepsy is a new field. Up until now, its mechanism has not been clear. In this study, a current condition neuromodulation platform was established for the quantitative measurement of iEEG signal and shunt-current density, and for building a current conduction pathway. The performance of the circuit system has been tested in vitro and the function of platform has been tested in vivo.

The implantable electrode tube included two working electrodes, one ground/reference electrode and a conduction electrode, that were fabricated and tested in vitro. The EIS of the implantable electrode tube showed good AC impedance characteristics, and was able to collect neural signals and conduct a current. On-bench system characterization provided evidence that the system had an excellent detection capability for weak signals compared to [29] and high current measurement accuracy for shunt-current quantization. The customized compact circuit was capable of measuring dual-channel iEEG signals with a relatively low noise floor, the shunt-current intensity was adjustable, and the current conduction path for current conduction treatment of epilepsy was investigated and validated with a short-term animal experiment.

Compared to traditional constant voltage or current stimulation [5], current flowing through a path to the outside of the brain to treat a seizure is new approach that was studies in this paper. Seizure induction by KA was detected through iEEG monitoring, while the shunt-current was successfully conducted out of the brain and recorded using the developed system, which verified the possibility of treating epilepsy with current conduction.

The proposed experimental platform was a great help in confirming the dose-response relationship between conduction current intensity and a therapeutic effect in rats after seizure induction by KA during current conduction treatment. This research is an important step and will help lay a foundation for the introduction of current conduction therapy for TLE into clinical research as soon as possible. The device structure is similar to a stimulator. Thus, it has great potential for treating humans with TLE in the future once the effectiveness, stability, and safety of the platform has been verified by animal experiments. Future work will involve improving the performance of the system in terms of electrode fabrication, iEEG recording, and shunt-current measurement. Further investigation is also needed for the fabrication of finer neural electrode probes with low impedance; a hardware system with a lower noise floor, smaller volume and lower power; and a stable current conduction pathway.

## Figures and Tables

**Table 1:** Technical data for the neural electrode.

Electrodes (Numbers)	Material	Length	Diameter
Working electrodes (2)	Stainless-steel	0.1 mm	0.1 mm
Conduction electrode (1)		1 mm	0.8 mm
Reference/Ground electrode (1)		0.3 mm	0.1 mm

## Data Availability

Not applicable.
